# Self-Made Cyclodextrin Inclusion Complexes for Enhanced Mechanical Properties of Polycaprolactone Composites

**DOI:** 10.3390/polym17070834

**Published:** 2025-03-21

**Authors:** Yanji Yin, Xiaotong Wang, Jiayan Zhang, Wenyan Wang, Rui Han

**Affiliations:** Key Laboratory of Materials and Surface Technology (Ministry of Education), School of Materials Science and Engineering, Engineering Research Center of Intelligent Air-Ground Integration Vehicle and Control, Xihua University, Chengdu 610039, China; yinkk99@126.com (Y.Y.); 0120240106@xhu.edu.cn (X.W.); zjy13060056505@163.com (J.Z.)

**Keywords:** polycaprolactone, cyclodextrin, inclusion complexes, mechanical property

## Abstract

Polycaprolactone (PCL) is a widely used polymer in biomedical applications due to its excellent processability, ductility, and biodegradability. However, single-component PCL devices often fail to meet the multifunctional and high-performance demands of modern biomedical devices. In this study, we introduced biodegradable and environmentally friendly cyclodextrin (CD) to fabricate CD inclusion complexes with low-molecular-weight polycaprolactone (LPCL). These complexes were incorporated into commercial PCL to prepare the resulting composites. The effects of PCL molecular weight and CD type on the properties and inclusion ratios of the complexes were systematically investigated. Among the tested complexes, the one formed with LPCL (Mw = 8 × 10^4^) and α-CD exhibited the highest inclusion ratio (2:1), significantly enhancing the crystallization and mechanical properties of the resulting composite (PCL/8-α-CD). Compared to pure PCL, the PCL/8-α-CD composite shows a 14.8% increase in crystallinity, along with improvements of 63.6% and 30.9% in tensile strength and elongation at break, respectively. These results demonstrate that the incorporation of CD inclusion complexes can effectively enhance the mechanical performance of PCL, making it a promising candidate for high-performance biomedical applications.

## 1. Introduction

Poly(ε-caprolactone) (PCL) is a widely used biodegradable polyester in biomedical applications due to its excellent processability, ductility, and biocompatibility [1,2,3,4]. It has been extensively employed in tissue engineering scaffolds [5], drug delivery systems [6], and sutures [7,8]. However, despite its favorable properties, single-component PCL devices often suffer from intrinsic limitations, such as low melting points and insufficient mechanical strength, which restrict their broader application in high-performance biomedical devices [9,10,11]. To address these limitations, significant efforts have been made to enhance the mechanical properties of PCL through the incorporation of fillers, blending with other polymers, or chemical modification [12,13].

Among various strategies, the use of cyclodextrin (CD) as a modifier has shown great promise due to its unique ability to form inclusion complexes with polymers. CD, a semi-natural product derived from starch, is cost-effective, non-toxic, biodegradable, and capable of forming host–guest inclusion complexes with a wide range of molecules [14,15,16]. Its conical cylindrical structure, with a hydrophobic cavity and hydrophilic exterior, enables the encapsulation of polymer chains, leading to enhanced intermolecular interactions and improved material properties [17,18,19]. Previous studies have demonstrated that CD can influence the crystallization behavior and mechanical performance of polymers. For instance, Khodaverdi et al. [20] developed a PCL-PEG-PCL triblock copolymer/CD supramolecular hydrogel for sustained drug release, while Aytac et al. [21] fabricated vitamin E/CD inclusion complexes encapsulated in PCL nanofibers for antioxidant delivery. Despite these advancements, challenges remain in achieving uniform dispersion of CD in PCL matrices, particularly in commercial-grade PCL, which is critical for realizing consistent and scalable performance enhancements [22].

In this work, we propose a novel approach to overcome these challenges by pre-forming CD inclusion complexes using low-molecular-weight polycaprolactone (LPCL) before incorporating them into commercial PCL. This strategy aims to improve the dispersion of CD in the PCL matrix and enhance the interfacial interactions between CD and PCL chains. LPCL, with its shorter and more flexible chains, can more easily form stable inclusion complexes with CD, ensuring a homogeneous distribution of CD within the commercial PCL matrix. This approach not only addresses the issue of CD dispersion but also optimizes the host–guest interactions between CD and PCL, leading to enhanced crystallization behavior and mechanical properties. Furthermore, we utilize micro-injection molding to fabricate high-performance PCL/CD composites. Micro-injection molding offers several advantages, including precise control over material dimensions, high production efficiency, and the ability to produce complex geometries, making it an ideal technique for manufacturing biomedical micro-devices [23,24,25].

Among the tested complexes, the one formed with LPCL (Mw = 8 × 10^4^) and α-CD exhibited the highest inclusion ratio (2:1), and this complex significantly enhances the crystallization and mechanical properties of the resulting composite (PCL/8-α-CD). Compared to pure PCL, the PCL/8-α-CD composite shows a 14.8% increase in crystallinity, along with improvements of 63.6% and 30.9% in tensile strength and elongation at break, respectively. These results demonstrate that the incorporation of CD inclusion complexes can effectively enhance the mechanical performance of PCL, paving the way for their application in high-performance biomedical devices. This study not only provides a new strategy for improving the properties of commercial PCL but also highlights the potential of CD-based modification techniques in the development of advanced polymer composites.

## 2. Experimental Section

### 2.1. Materials

α-Cyclodextrin (α-CD, CAS: 10016-20-3, purity ≥ 99.0%), β-cyclodextrin (β-CD, CAS: 7585-39-9, purity ≥ 99.0%), and γ-cyclodextrin (γ-CD, CAS: 17465-86-0, purity ≥ 98.0%) were purchased from Adamas Reagent Co., Ltd. (Shanghai, China). Polycaprolactone (PCL, CAS: 24980-41-4, analytical grade) was obtained from Solvay (Brussels, Belgium). The solvents used in this study, including N,N-dimethylformamide (DMF, analytical grade), dimethyl sulfoxide (DMSO, purity ≥ 99.0%), and acetone (purity ≥ 99.0%), were also supplied by Adamas Reagent Co., Ltd. Deionized water was provided by Chengdu Cologne Chemical Co., Ltd. (Chengdu, China). 

### 2.2. Sample Fabrication

#### 2.2.1. Preparation of CD Inclusion Complexes Using Low-Molecular-Weight PCL

β-CD, α-CD, and γ-CD were vacuum-dried at 80 °C for 24 h, while low-molecular-weight PCL (LPCL, Mw = 8 × 10^4^ and 5 × 10^4^) was vacuum-dried at 40 °C for 24 h. A predetermined mass of CD was accurately weighed and dissolved in 10 mL of DMF, resulting in a colorless, transparent solution under constant stirring at 50 rpm using a magnetic stirrer maintained at 50 °C. Simultaneously, 0.4 g of LPCL was dissolved in 60 mL of acetone under the same stirring conditions to obtain a clear, transparent solution. The CD solution was then added dropwise to the LPCL solution at a constant rate. The resulting mixture was stirred at 50 °C for 3 h and subsequently cooled to room temperature while continuously stirring for an additional 12 h. The final product was rinsed three times with deionized water and dried at 40 °C in an oven. The complexes were labeled as 5-α-CD, 5-β-CD, 5-γ-CD, 8-α-CD, 8-β-CD, and 8-γ-CD, where “5” and “8” correspond to LPCL with molecular weights of 5 × 10^4^ and 8 × 10^4^, respectively. The specific compositions are detailed in Table 1.

#### 2.2.2. Preparation of the Resulting PCL Composites

**Figure 1:**
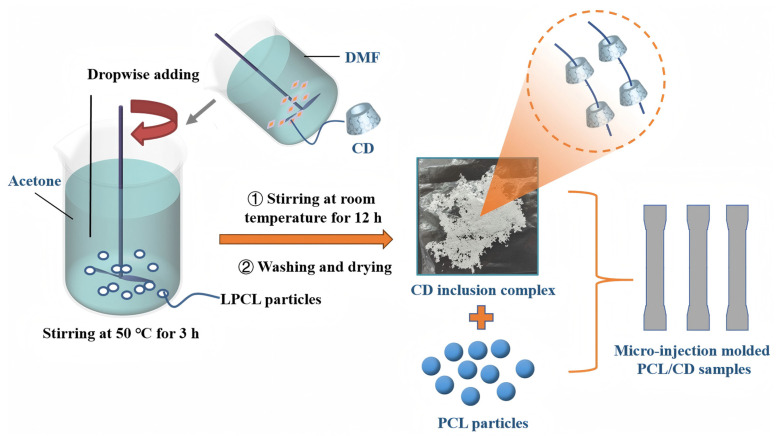
Fabrication process of the resulting PCL composites including the preparation of CD inclusion complexes.

Prior to micro-injection molding, the machine cavity was thoroughly cleaned with PCL to ensure a contamination-free environment. Based on the structural characterization and analysis of the inclusion complexes, α-CD was selected for modifying commercial PCL due to its superior inclusion ratio and compatibility. The pre-formed 8-α-CD and 5-α-CD inclusion complexes were then added to commercial PCL at a total loading of 20 wt%. The micro-injection molding process was carried out at a temperature of 80 °C and an injection speed of 100 mm/s. The resulting molded specimens had dimensions of 9 mm × 2 mm × 0.2 mm. And the resulting composites are labeled as PCL/8-α-CD and PCL/5-α-CD. The fabrication processes of PCL-CD inclusion complexes and the micro-injection molded samples are shown in Figure 1.

### 2.3. Characterization

The chemical structure and morphology of LPCL, inclusion complexes, and PCL/α-CD composites were characterized using scanning electron microscopy (SEM, JSM-IT800, Tokyo, Japan). Prior to imaging, samples were sputter-coated with gold for 90 s and observed at an accelerating voltage of 15 kV. Fourier transform infrared spectroscopy (FTIR, Nicolet 6700, Thermo Scientific, Waltham, MA, USA) was employed to analyze the structural features of the samples. Nuclear magnetic resonance hydrogen spectroscopy (^1^H NMR, Varian Unity Inova 400, Agilent Technologies, Santa Clara, CA, USA) was conducted using deuterated chloroform (CDCl_3_) and deuterated dimethyl sulfoxide (DMSO-d_6_) as solvents for PCL and PCL/CD composites, respectively, at a radio frequency of 400 MHz. The crystal structure of the samples was examined by X-ray diffraction (XRD) with a scanning range of 5° to 60° (Cu Kα radiation, λ = 0.154 nm) at a scanning rate of 4°/min.

The crystallization behavior of the samples was observed using a hot-stage polarizing microscope (POM). Samples were heated from room temperature to 90 °C at a rate of 30 °C/min, held for 5 min to eliminate thermal history, and then cooled to 45 °C at 30 °C/min, where crystallization images were captured after holding for 20 min. Differential scanning calorimetry (DSC, Q20, TA Instruments, New Castle, DE, USA) was used to analyze the melting and crystallization behaviors of the samples over a temperature range of 0 °C to 80 °C at a heating or cooling rate of 10 °C/min. The dynamic viscoelastic behavior was measured using a dynamic rheometer (AR2000ex, TA Instruments, USA) with a 25 mm parallel plate fixture and a 1 mm gap in frequency sweep mode. Tests were performed at 80 °C with a strain of 1% and a frequency range of 0.01–100 rad/s.

The dynamic mechanical properties of the micro-injection molded samples were evaluated using dynamic mechanical analysis (DMA, Q800, TA Instruments, USA) in tensile mode. The temperature range was set from −100 °C to 40 °C at a heating rate of 3 °C/min and a frequency of 1 Hz. Tensile properties were tested according to the GB/T1040.2-2006 standard [26] using a universal testing machine (5576, Instron, Norwood, MA, USA). Dumbbell-shaped specimens with dimensions of 9 mm × 2 mm × 0.2 mm were tested at a tensile speed of 10 mm/min at room temperature.

## 3. Results and Discussion

### 3.1. Structure and Crystal Analysis of CD Inclusion Complexes

**Figure 2:**
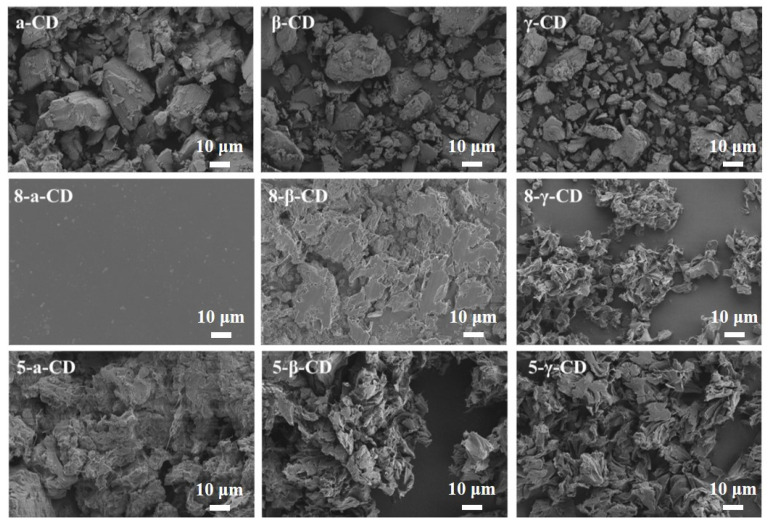
SEM of different CD and CD inclusion complexes.

The surface morphologies of CD inclusion complexes containing LPCL (Mw = 5 × 10^4^ and 8 × 10^4^) were examined using SEM, as shown in Figure 2. CD exhibits a powdery morphology with blocky particles. The inclusion complexes formed with different CDs and LPCL display distinct morphologies. The 8-α-CD complex shows a smooth, film-like surface. In contrast, 8-β-CD exhibits increased surface roughness with a continuous blocky structure, while 8-γ-CD displays a dispersed flocculent structure. When the molecular weight of LPCL is 5 × 10^4^ the 5-α-CD complex appears as continuous blocks with visible filamentous connections, whereas 5-β-CD and 5-γ-CD both show a dispersed flocculent structure without filamentous features. These results indicate that both the molecular weight of LPCL and the type of CD significantly influence the micro-morphology of the inclusion complexes, likely due to differences in inclusion efficiencies between various CDs and LPCL molecular weights.

**Figure 3:**
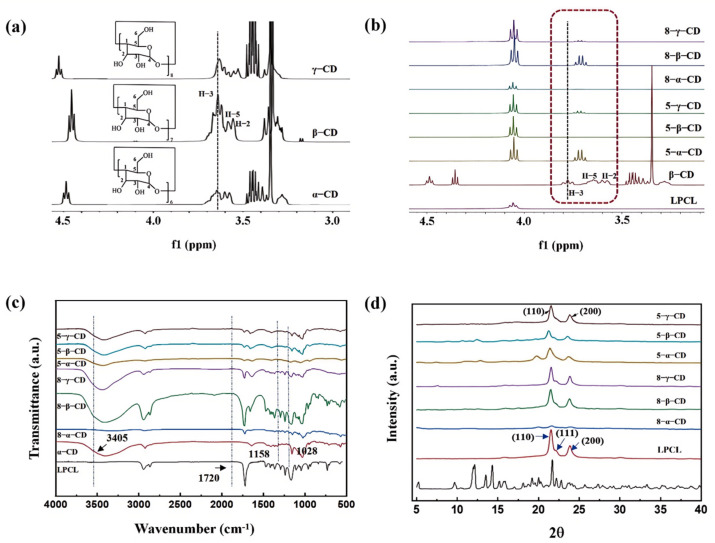
(**a**) ^1^H NMR of α-CD, β-CD, and γ-CD, (**b**) ^1^H NMR of CD inclusion complexes, (**c**) FTIR spectrum, and (**d**) XRD curves of CD inclusion complexes.

The changes in the proton environments of LPCL and CD before and after inclusion were investigated using ^1^H NMR spectroscopy, as shown in Figure 3a,b. For LPCL, the triplet peak at 4.06 ppm corresponds to the −CH_2_ group adjacent to the -O- group. After inclusion, the coexistence of PCL’s characteristic peak at 4.06 ppm and CD’s characteristic peaks confirmed the presence of both CD and LPCL in the inclusion complexes. The protons inside the CD cavity (H-3 and H-5) are more sensitive to complexation than those outside the cavity (H-1, H-2, and H-4) [27,28,29]. The upfield shift in the H-3 and H-5 signals in CD indicated the formation of host–guest interactions between PCL and CD [30,31]. Based on the area ratios of the characteristic peaks in the ^1^H NMR spectra, the inclusion ratios of the different complexes were calculated. The results revealed molar ratios of PCL:CD for 5-α-CD, 5-β-CD, 5-γ-CD, 8-α-CD, 8-β-CD, and 8-γ-CD to be 3:1, 5:1, 68:1, 2:1, 5:1, and 6:1, respectively. These findings demonstrate that a smaller CD cavity size and a higher PCL molecular weight favor improved inclusion efficiency, leading to higher inclusion ratios. Among the complexes, 8-α-CD exhibited the highest inclusion ratio of 2:1. This high inclusion efficiency can be attributed to the smaller cavity size of α-CD, which allows for stable and orderly nesting along the LPCL molecular chain, minimizing slippage. Additionally, the longer molecular chains of LPCL (Mw = 8 × 10^4^) provide a more favorable environment for the stable nesting of CD.

The chemical structure characteristics of the CD inclusion complexes were investigated using FTIR spectroscopy, as illustrated in Figure 3c. In the FTIR spectrum of CD, a strong O-H stretching vibration peak is observed at 3405 cm⁻^1^, along with a −CH_2_− stretching vibration peak at 2920 cm⁻^1^ [32]. The peak at 1632 cm⁻^1^ is attributed to the bending vibration of CH_2_. For PCL, the carbonyl (C=O) stretching band appears at 1720 cm⁻^1^, while the stretching vibrations of −CH and −CH_2_− are detected in the region of 2876–2943 cm⁻^1^ [33,34]. In the CD inclusion complexes, the characteristic peaks of both LPCL and CD are clearly visible, confirming the successful incorporation of CD into the LPCL matrix. However, due to the overlap of characteristic peaks between CD and PCL, some CD-specific peaks could not be clearly identified. Notably, the carbonyl stretching band of LPCL exhibits a slight shift toward higher wavenumbers (indicating an amorphous tendency) and a significant suppression in intensity, suggesting the formation of inclusion complexes with CD [31,35,36]. Furthermore, the −OH peak of CD at 3405 cm⁻^1^ shifts to a higher wavenumber in the complexes, providing additional evidence for the host–guest interactions between CD and LPCL.

The XRD spectra of various samples are illustrated in Figure 3d. For LPCL, three distinct diffraction peaks are observed at 21.5°, 22.0°, and 23.8°, corresponding to the (110), (111), and (200) crystal planes [34,37,38,39], respectively. A diffuse peak between 15° and 25° indicates a broad distribution of interplanar spacing, suggesting the coexistence of crystalline and amorphous phases in LPCL. These characteristic diffraction peaks are also present in all CD inclusion complexes. Notably, the 5-α-CD complex, prepared with LPCL (Mw = 5 × 10^4^) and α-CD, exhibits a new diffraction peak at 19.6°, indicating the formation of a new crystal type. This phenomenon can be attributed to the penetration of LPCL molecular chains into the α-CD cavities, forming inclusion complexes that influence LPCL crystallization and lead to a tunnel-like crystal arrangement [32,40,41]. Additionally, although the peak intensity of 8-α-CD was weaker, the characteristic peaks of LPCL at 21.5° and 23.8° are still observed, along with a new peak at 19.8°, confirming the formation of the 8-α-CD inclusion complex. Overall, the XRD results provide compelling evidence for the significant host–guest interactions between LPCL and α-CD, highlighting the impact of α-CD on the crystallization behavior of LPCL.

**Figure 4:**
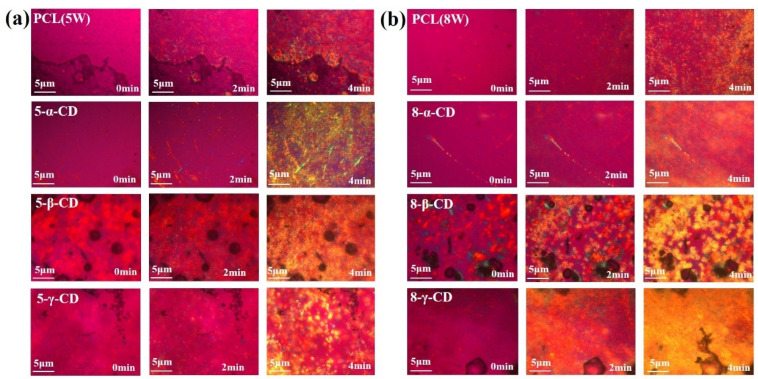
POM diagrams of isothermal crystallization of different inclusions at 42 °C, where the molecular weights of LPCL in the inclusions are 5 × 10^4^ (5 W) (**a**) and 8 × 10^4^ (8 W) (**b**), respectively.

The crystallization process and crystalline morphology of the inclusion complexes were further examined using polarizing microscopy (POM). Figure 4 shows the POM images of various CD inclusion complexes during isothermal crystallization at 42 °C. Polymer crystallization generally involves two stages: nucleation and growth [42,43,44]. The nucleation rate is influenced by the nucleation energy barrier, while the growth rate is controlled by the diffusion and regular stacking of molecular chains [44,45]. For pure LPCL (Mw = 8 × 10^4^ and 5 × 10^4^), the isothermal crystallization exhibits typical spherulitic morphologies. However, upon the addition of α-CD, both LPCL types display additional columnar crystal morphologies, which can be attributed to the nesting of α-CD on LPCL chains. This observation confirms the presence of host–guest interactions between α-CD and LPCL. In contrast, no distinct columnar crystals were observed in the β-CD and γ-CD inclusion complexes, likely due to their lower nesting ratios, consistent with the ^1^H NMR results. Interestingly, the 5-β-CD and 8-γ-CD complexes show an increased number of crystal nuclei and faster crystal growth rates (Figure 4a,b). This behavior can be explained by the relatively low nesting ratios of β-CD and γ-CD, which primarily act as heterogeneous nucleating agents, promoting LPCL nucleation. Additionally, the reduced constraint from CD allows LPCL molecular chains to diffuse more freely, leading to enhanced crystallization rates.

In conclusion, the POM results, supported by ^1^H NMR data, provide clear evidence of the formation of inclusion complexes in 5-α-CD and 8-α-CD. Based on these findings, α-CD is selected for further investigation due to its superior ability to form stable inclusion complexes with LPCL.

### 3.2. Structure and Crystal Analysis of PCL/α-CD Composites

**Figure 5:**
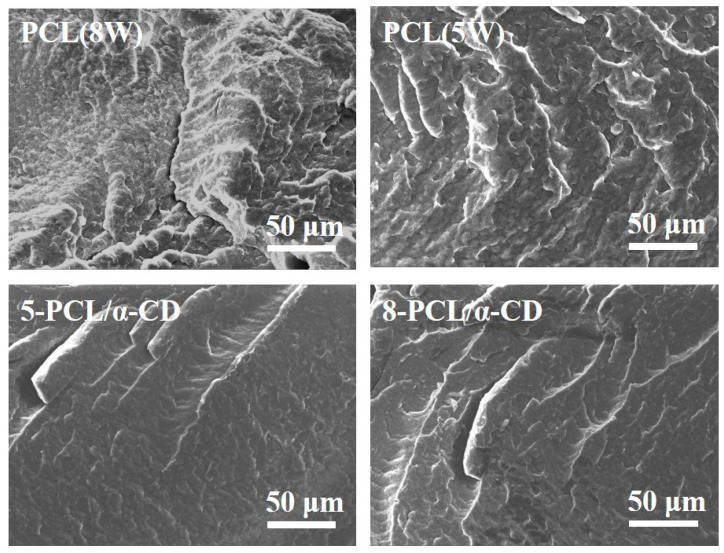
SEM images of brittle section for PCL and its composites.

To investigate the influence of the microstructure on the mechanical properties of PCL/8-α-CD and PCL/5-α-CD composites, the brittle fracture surfaces of these materials were examined using SEM, as shown in Figure 5. Under identical fracture conditions, the fracture surfaces of pure PCL samples exhibit a rugged morphology, indicative of their good toughness. However, after the addition of 5-α-CD and 8-α-CD, the fracture surfaces become significantly smoother, suggesting an increase in hardness. This change can be attributed to the formation of inclusion complexes, which restrict the movement of PCL chains, thereby enhancing the material’s hardness. Importantly, no obvious heterogeneous structures are observed in the fracture surfaces of the PCL/α-CD composites, indicating excellent compatibility and uniform dispersion of the inclusion complexes within the PCL matrix.

**Figure 6:**
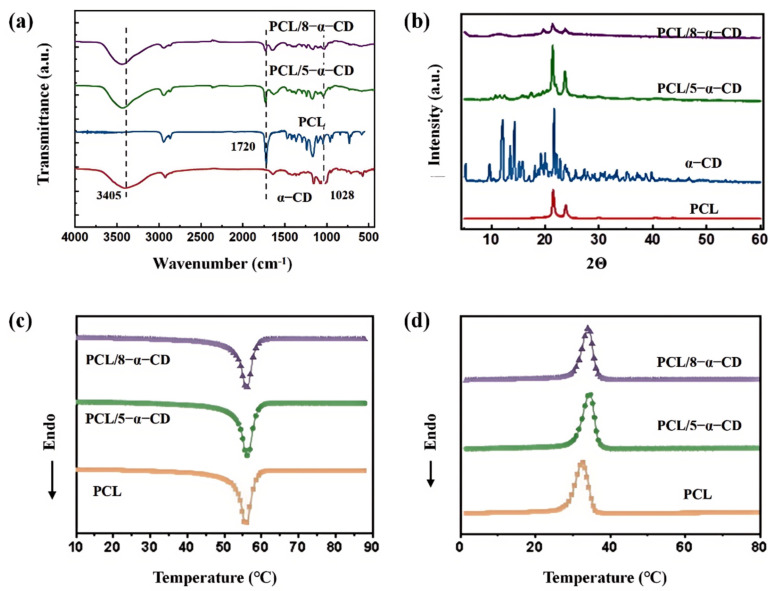
(**a**) Infrared spectra, (**b**) XRD analysis, (**c**) DSC melting, (**d**) cooling curves of different samples.

The chemical structures of the PCL/α-CD composites were characterized using FTIR spectroscopy, as shown in Figure 6a. Similar to the inclusion complexes (Figure 3c), the FTIR spectra of PCL/8-α-CD and PCL/5-α-CD display distinct hydroxyl peaks of α-CD and carbonyl peaks of PCL. Notably, the carbonyl peak of PCL shifts to higher wavenumbers, and its intensity is suppressed, indicating that the CD remained attached to the PCL chains even under the high-speed shearing conditions of micro-injection molding. The crystallization properties of the PCL/α-CD composites are further analyzed using XRD, as illustrated in Figure 6b. Both PCL/5-α-CD and PCL/8-α-CD exhibit diffuse crystallization peaks of α-CD (10–20°) and the characteristic diffraction peaks of PCL (21.6° and 23.9°), confirming the presence of α-CD in the composites. Notably, PCL/8-α-CD shows a new crystallization peak at 19.8°, consistent with the 8-α-CD inclusion complex (Figure 3d), indicating the formation of a new crystal type. In contrast, the new peak observed in the 5-α-CD inclusion complex (Figure 3d) is absent in PCL/5-α-CD. This can be attributed to the shorter LPCL chains (Mw = 5 × 10^4^) in the 5-α-CD complex, which lead to partial detachment of CD from the PCL chains under high-speed shearing. As a result, the influence of 5-α-CD on the crystallization process is weakened, making the new crystal type difficult to detect in PCL/5-α-CD.

The thermal behavior of the micro-injection molded specimens of PCL, PCL/8-α-CD, and PCL/5-α-CD was investigated using DSC, as shown in Figure 6c,d. The relevant thermal parameters, including Tc, Tm, Xc, and crystallization enthalpy, are summarized in Table 2. Both PCL/8-α-CD and PCL/5-α-CD composites exhibit melting transition processes similar to that of pure PCL. However, the crystallization temperature of PCL in the composites increases by approximately 2 °C, and the crystallinity improves by about 14.8%, indicating that the addition of 8-α-CD and 5-α-CD significantly enhanced the crystallization behavior and crystalline integrity of PCL. Actually, the high-speed shearing field during micro-injection molding promotes the thermal motion of PCL molecular chains and facilitates the orientation of chain segments into a more regular arrangement. Particularly, the inclusion complexes further strengthen intermolecular interactions, enabling the regularly arranged segments to form stable nuclei at lower subcooling temperatures. As a result, PCL/α-CD crystallizes at a higher temperature, leading to increased crystallinity and improved crystal integrity. These findings highlight the effectiveness of α-CD inclusion complexes in enhancing the thermal and crystallization properties of PCL.

### 3.3. Rheological Behavior Analysis of PCL/α-CD Composites

**Figure 7:**
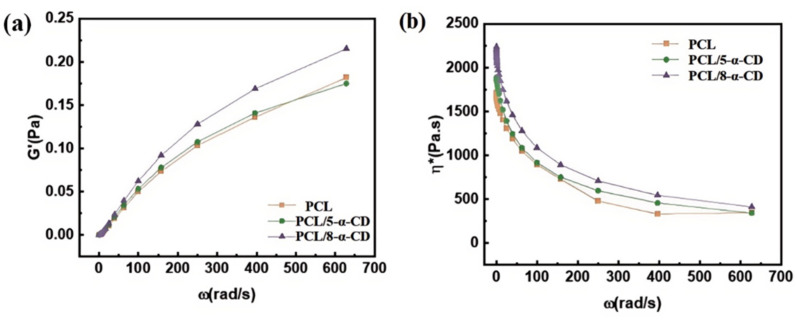
Rheological behavior of different samples in (**a**) storage modulus and (**b**) viscosity.

The rheological properties of polymer composites differ significantly from those of pure polymers, reflecting the intermolecular interactions within the composites. Figure 7a,b show the rheological curves of storage modulus (G’) and complex viscosity (|η*|) for PCL, PCL/8-α-CD, and PCL/5-α-CD, obtained through small amplitude oscillatory scanning at 80 °C. As the shear frequency increases, G’ exhibits an upward trend, while |η*| decreases, consistent with the shear-thinning behavior typical of pseudoplastic fluids. The addition of α-CD inclusion complexes results in a higher storage modulus and complex viscosity compared to pure PCL, indicating enhanced intermolecular interactions in the PCL/α-CD composites. This enhancement can be attributed to the formation of physical crosslinks and increased hindrance to molecular chain movement, which improve the elasticity and viscosity of the composites. Notably, PCL/8-α-CD exhibits higher G’ and |η*| values than PCL/5-α-CD, suggesting that 8-α-CD is more effective in strengthening intermolecular interactions within PCL.

### 3.4. Mechanical Property of PCL/α-CD Composites

**Figure 8:**
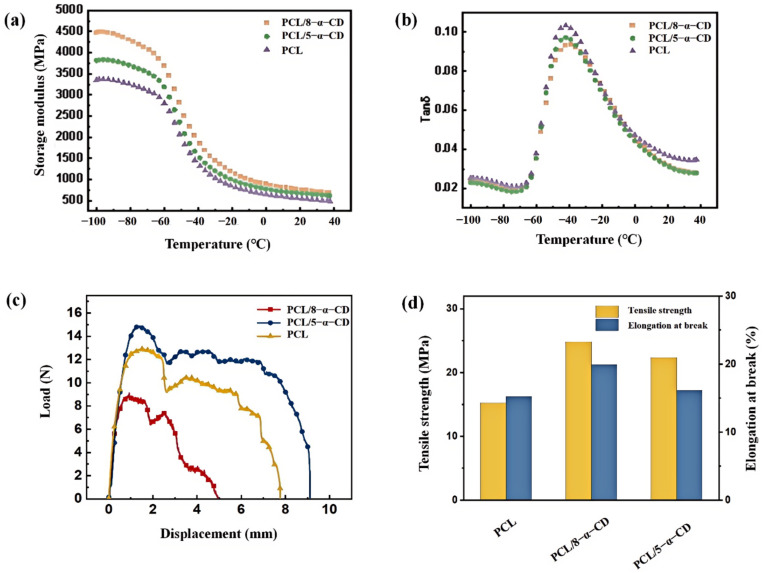
DMA curves of different samples, (**a**) storage modulus, (**b**) loss factor, (**c**,**d**) tensile curves and tensile properties of different samples at 10 mm/min tensile rate.

To investigate the influence of different inclusion complexes on the mechanical properties of PCL/α-CD composites, we measured the dynamic mechanical properties and tensile properties of these composites (Figure 8). Figure 8a,b show the storage modulus (G’) and loss factor (Tanδ) curves for different samples in the range of −100–40 °C. G’ reflects the material’s ability of energy storage and deformation resistance, while Tanδ represents the material’s viscous character. As shown in Figure 8a, the storage modulus (G’) of all samples decreases with increasing temperature, exhibiting an inverse S-shaped trend. The storage modulus of both PCL/8-α-CD and PCL/5-α-CD composites is higher than that of PCL, indicating that inclusion complexes were able to enhance the stiffness of PCL. This is rooted in the enhanced intermolecular interactions due to the addition of α-CD, resulting in increased stiffness. Moreover, the G’ increase in PCL/8-α-CD is more significant than that of PCL/5-α-CD. As previously analyzed, PCL with a longer chain has a higher nesting rate, leading to stronger constraints on the chain segments, which hinders the deformation of the PCL matrix, resulting in the higher stiffness of PCL/8-α-CD. Additionally, the bowl-shaped structure of CD on PCL chains provides protection from external stress and improves the storage modulus. Figure 8b and Table 3 illustrate the trend of loss factor (Tanδ) with increasing temperature and the glass transition temperature (Tg) of the samples. Prior to the glass transition, all samples are in the glassy state, where chain segments are restricted, showing low mechanical loss. As the temperature increases, the amorphous regions of the system transfer from a glassy to a rubbery state, and chain segments begin to move, leading to a sharp increase in internal friction and Tanδ values. Additionally, Table 3 shows that compared to PCL (−43.2 °C), the Tg of both PCL/8-α-CD (−39.9 °C) and PCL/5-α-CD (−41.9 °C) rose. It is known that Tg reflects the transition of polymer chain segments in the amorphous regions from frozen to a mobile state. The addition of inclusion complexes restricts the movement of PCL chain segments, which caused the raising in activation energy for chain segment movement, resulting in an elevated Tg. After the glass transition, the Tanδ values of PCL/α-CD composites are lower than that of PCL, denoting that the introduction of inclusion complexes can reduce internal friction, achieving a reduced damping performance.

The force–displacement curves and tensile properties of the micro-injection molded specimens are illustrated in Figure 8c,d and Table 4. The addition of inclusion complexes significantly enhances the tensile strength and elongation at the break of PCL. Specifically, PCL/8-α-CD exhibits the highest tensile strength (24.8 MPa) and elongation at break (19.9%), representing increases of 63.2% and 30.9%, respectively, compared to pure PCL. The improvement in tensile strength can be primarily attributed to the “molecular necklace” structure formed by the inclusion complexes, which increases nucleation sites for PCL, enhances crystal integrity, and leads to higher crystallinity. Additionally, the inclusion complexes strengthen intermolecular interactions, facilitating more efficient stress transfer within the material, thereby improving both tensile strength and elongation at break. In contrast, PCL/5-α-CD shows lower tensile strength and elongation at break compared to PCL/8-α-CD. This is mainly due to the lower nesting efficiency of 5-α-CD, which limits its ability to enhance intermolecular interactions and crystallinity in PCL. The longer molecular chains of PCL (Mw = 8 × 10^4^) in PCL/8-α-CD provide stronger constraints on chain segments, resulting in a more stable and ordered structure that better resists deformation under stress. Furthermore, the bowl-shaped structure of CD may offer additional protection against external stress, contributing to the improved mechanical performance of PCL/8-α-CD. These findings highlight the critical role of α-CD inclusion complexes in enhancing the mechanical properties of PCL for high-performance applications.

## 4. Conclusions

In this study, CD inclusion complexes were prepared using LPCL (Mw = 8 × 10^4^ and 5 × 10^4^) and CDs (α-CD, β-CD, and γ-CD). The 5-α-CD and 8-α-CD complexes exhibit the highest inclusion ratios and crystallization properties, making them ideal for enhancing PCL performance. These complexes were incorporated into commercial PCL to fabricate micro-injection molded composites, which were systematically characterized. The results show that α-CD and higher molecular weight LPCL (Mw = 8 × 10^4^) formed stable inclusion complexes with enhanced intermolecular interactions, improving crystallization behavior and crystallinity. Mechanically, the PCL/8-α-CD composite exhibited the highest tensile strength (24.8 MPa) and elongation at break (19.9%), representing increases of 63.6% and 30.9%, respectively, over pure PCL. These improvements are attributed to the formation of a “molecular necklace” structure, which enhances intermolecular interactions and stress transfer. This structure not only strengthens the material but also improves its overall mechanical performance. In summary, the incorporation of α-CD inclusion complexes significantly improved the crystallization, tensile strength, and toughness of PCL, demonstrating its potential for high-performance micro-injection molded parts. Future work could explore applications in biomedical devices, such as vascular clips or bone screws.

## Figures and Tables

**Table 1:** The composition of CD inclusion complexes.

Sample	LPCL/α, β, γ-CD (mol/mol)	LPCL/Acetone (g/mL)	A, β, γ-CD/DMF (g/mL)
5-α-CD	1/40	0.250/60	0.1946/10
5-β-CD	1/40	0.250/60	0.227/10
5-γ-CD	1/40	0.250/60	0.2594/10
8-α-CD	1/40	0.400/60	0.1946/10
8-β-CD	1/40	0.400/60	0.227/10
8-γ-CD	1/40	0.400/60	0.2594/10

**Table 2:** DSC melt crystallization parameters of different samples.

Sample	T_c_/°C	T_m_/°C	X_c_%	Heat Flow (w/g)
PCL	32.55	55.74	39.3	49.45
PCL/5-α-CD	34.44	56.18	44.6	55.452
PCL/8-α-CD	34.12	56.01	45.1	50.214

**Table 3:** Glass transition temperature of different samples.

Sample	Glass Transition Temperature Tg (°C)
PCL	−43.2
PCL/5-α-CD	−41.9
PCL/8-α-CD	−39.9

**Table 4:** Tensile property parameters of different samples.

Sample	Tensile Strength (MPa)	Elongation at Break (%)
PCL	15.2	15.2
PCL/8-α-CD	24.8	19.9
PCL/5-α-CD	22.3	16.1

## Data Availability

The original contributions presented in this study are included in the article. Further inquiries can be directed to the corresponding authors.
